# When to trust our learners? Clinical teachers’ perceptions of decision variables in the entrustment process

**DOI:** 10.1007/s40037-018-0430-0

**Published:** 2018-04-30

**Authors:** Chantal C. M. A. Duijn, Lisanne S. Welink, Harold G. J. Bok, Olle T. J. ten Cate

**Affiliations:** 1 Center for Quality Improvement in Veterinary Education, Faculty of Veterinary Medicine, Utrecht University, Utrecht, The Netherlands; 2 Center for Research and Development of Education, University Medical Center Utrecht, Utrecht, The Netherlands

**Keywords:** Clinical teachers, Decision variables, Entrustable professional activities, EPAs, Workplace-based assessment, Focus group, Supervision level

## Abstract

**Introduction:**

Clinical training programs increasingly use entrustable professional activities (EPAs) as focus of assessment. However, questions remain about which information should ground decisions to trust learners. This qualitative study aimed to identify decision variables in the workplace that clinical teachers find relevant in the elaboration of the entrustment decision processes. The findings can substantiate entrustment decision-making in the clinical workplace.

**Methods:**

Focus groups were conducted with medical and veterinary clinical teachers, using the structured consensus method of the Nominal Group Technique to generate decision variables. A ranking was made based on a relevance score assigned by the clinical teachers to the different decision variables. Field notes, audio recordings and flip chart lists were analyzed and subsequently translated and, as a form of axial coding, merged into one list, combining the decision variables that were similar in their meaning.

**Results:**

A list of 11 and 17 decision variables were acknowledged as relevant by the medical and veterinary teacher groups, respectively. The focus groups yielded 21 unique decision variables that were considered relevant to inform readiness to perform a clinical task on a designated level of supervision. The decision variables consisted of skills, generic qualities, characteristics, previous performance or other information. We were able to group the decision variables into five categories: ability, humility, integrity, reliability and adequate exposure.

**Discussion:**

To entrust a learner to perform a task at a specific level of supervision, a supervisor needs information to support such a judgement. This trust cannot be credited on a single case at a single moment of assessment, but requires different variables and multiple sources of information. This study provides an overview of decision variables giving evidence to justify the multifactorial process of making an entrustment decision.

## What this paper adds

Clinical training programs increasingly use entrustable professional activities (EPAs) as focus of assessment, but questions remain about which information should ground decisions to trust learners. Clinical teachers struggle to find ways to assess the preparedness of learners to independently perform patient care tasks. An entrustment decision cannot be credited to a single variable but requires a multitude of variables. We defined decision variables as the characteristics, skills, qualities, previous performance or other information about learners that provide supervisors with input about learners’ performance on an EPA to make valid entrustment decisions. Such decision variables include features that determine the learners’ trustworthiness, the clinicians’ trust propensity and the risks and benefits involved in entrustment decisions. This study provides an overview of decision variables that clinical teachers find relevant in the elaboration of the entrustment decision processes into five categories: ability, humility, integrity, reliability and adequate exposure. The findings can substantiate entrustment decision-making in the clinical workplace. Extensive reflection by different observers on performed tasks is highly relevant: both reviewing of actions after shifts by teachers, reflection by the learner her or himself as well as multi-source feedback were mentioned as relevant decision variables to base entrustment decisions on. Faculty development will be necessary to explain these types of entrustment decisions as being situational versus focused on summative assessment when introducing workplace curricula based on EPAs.

## Introduction

Assessing medical learners in the workplace is notoriously difficult [1–3]. Clinical teachers struggle to find meaningful ways to assess the preparedness of learners to independently perform patient care tasks [4]. Assessment often relies on subjective impressions of clinical teachers, who are often not specifically trained in assessing learners [5, 6]. The ultimate purpose of assessment is typically not to know how learners have acted in the past, but to predict how they will act in the near future [7]. One way to improve medical learner assessment may be reframing assessment to the question: ‘(When) can we trust this learner to execute this critical task with a certain level of supervision?’. In this approach, the concept of trust is crucial [8–11].

Using entrustable professional activities (EPAs)—units of professional practice to be entrusted to learners once they have demonstrated sufficient competence—in medical training programs requires elaboration of entrustment decision processes [11–14]. Entrustment decisions require a grounded entrustment process, based on decision variables collected in the clinical workplace over time. These decision variables should provide evidence to determine the learner’s trustworthiness, the clinicians’ trust propensity and the risks and benefits involved in entrustment decisions [12, 15]. In this study decision variables are defined as any information (e. g. skills, generic qualities, characteristics or previous performance) that provides supervisors with input about learner performance on an EPA in order to make valid entrustment decisions.

Variables that affect the confidence to trust a learner in performing a clinical task at a certain level of supervision have been a topic of research in the past decade [4, 16–19]. The construct of ‘trusting a learner’ feels better aligned with how clinicians think than regular rating scales in workplace-based assessments [20–22]. Teachers often attend to or value different aspects of performance, lack a clear standard for judging performance or use variable standards against which to judge learners [23–25]. Assessors frequently compare learner performance with what they would do (self as standard) or to what they would expect of learners at a similar level of training; some rely on a gut feeling [23]. Clinical teachers regularly make judgements of learners’ competence at the point-of-care, in order to allow them to deliver patient care. They reported incorporating their perceptions of learners’ credibility and willingness to seek help when determining how much they trusted those learners [8]. In making these decisions about the level of supervision required in a given clinical situation, supervisors assess learners’ trustworthiness, a multidimensional construct which includes *clinical knowledge and skills, discernment of limitations, truthfulness,* and *conscientiousness* [16] and other features [8, 26]. These variables were recently reframed as: *ability* (knowledge, skill, experience) to complete the task, but also *integrity* (truthfulness, benevolence), *reliability* (conscientiousness, consistency), and *humility* (observing limits, willingness to ask for help) [27].

Entrustment decisions combine traditional assessment of ability with the right to execute an EPA without (or with indirect) supervision [23]. There are many variables of influence in determining how, when, and whether learners are granted responsibilities under indirect or distant supervision [7, 14]. Clinical teachers should take these variables into account and push learners to stretch their scope of performance with the need for safe, high-quality patient care. A previous study provided an overview of information sources derived from discussions with experts [11]. The aim of the current study is to identify the decision variables clinical teachers use and value in making entrustment decisions. Therefore, we addressed the following research question: Which decision variables are perceived by clinical teachers as being relevant to make entrustment decisions on learners’ progress towards unsupervised practice?

## Method

### Design

An exploratory, qualitative multidisciplinary and multi-profession study was performed using focus groups with clinical teachers from various specialties [28]. The consensus method of the nominal group technique was applied [29].

### Educational setting

Two focus groups were conducted involving clinical teachers in medical and veterinary education. The focus group with medical teachers was conducted at the University Medical Center, Utrecht University, the Netherlands (UMCU). The focus group with veterinary teachers was organized at the Faculty of Veterinary Medicine, Utrecht University, the Netherlands (FVMU). Veterinary and medical education are similar in that learners participate and learn in authentic clinical settings with real patients under direct supervision and are gradually granted increased responsibility to execute clinical tasks [30]. The clinical teachers are usually also involved in residency training programs.

### Participants

Participants were selected using purposeful sampling. They were selected because of their role in clinical teaching, their familiarity with the concept of EPAs and their experience in regularly supervising learners in the workplace. We deliberately selected clinical teachers from different specialties to ensure a heterogeneous group. A priori we determined a sample size of 8–10 participants per focus group, being recommended as an adequate group size [29]. All participants were sub-specialists with experience in supervising several learner cohorts (both undergraduates and postgraduates) in their specialty.

### Focus group procedure

To obtain in-depth information and motivate teachers to share their views, focus groups [28] were conducted (in Dutch), based on the structured consensus method of the nominal group technique (NGT) as described by O’Neil and Jackson [29]. NGT is a structured activity facilitating group-based decision-making controlled by a moderator. A moderated group discussion takes place until no new ideas are generated. The outcome of this process is a ranked list of ideas derived from the focus group sessions [29].

Each focus group session started with an introduction of the topic and the purpose of the meeting. After the introduction, a trigger case was presented, using an example of a critical EPA. For veterinary medicine, the example was a caesarean section in a cow [30], and for medical education a lumbar puncture [31]. After briefly introducing these trigger cases, two questions were projected on a screen:

1. Based on what information would you be able to make a valid entrustment decision about a learner’s preparedness to perform an EPA without direct supervision?
2. Who or what should provide this information and in which way?

The participants were instructed to use the trigger case, but not to restrict their ideas about decision variables to just this example. After posing the two main questions, participants were asked to write down their ideas in silence for about 5 minutes.

The second phase of the NGT consisted of each group member verbalizing one decision variable at a time. They took turns, each proposing one idea; participants were not allowed to react to previously stated ideas. This cycle continued until no new ideas emerged. Every item mentioned was directly written on a whiteboard or flip chart by the moderator, allowing the whole group to read a growing list of ideas and to be stimulated to think of further ideas. When necessary, a brief dialogue for clarification was conducted.

In the third phase, each of the participants was asked to create, in silence, a top 5 ranking (5 = highest priority and 1 = lowest priority) of their ideas. These rankings were averaged for all participants until a full list of ideas in order of priority was formed. During the focus group sessions, two observing investigators (LW and CD) took field notes. The two sessions were audio-recorded and transcribed verbatim to help to understand and retain the clinical teachers’ considerations.

### Researcher roles and backgrounds

The veterinary focus group session was facilitated by a medical education researcher (OtC), the medical focus group by a veterinary education researcher (HB). They had no personal or professional connection with the participants. Both facilitators were experienced moderators of focus groups. LW and CD were present as observers at both focus group sessions. FVMU participants were recruited by CD and HB. CD is a veterinarian working as a veterinary practitioner and a PhD candidate in veterinary education research. UMCU participant recruitment for the medical focus group was done by LW and OtC. LW is a recent graduate from the Utrecht medical school and was working as a junior educator at the time of this study.

### Data analysis

LW merged the data from the whiteboard or flipchart lists with the field notes and the transcripts to arrive at two lists of decision variables, one for each focus group. These lists were checked by CD. In a second phase, as a form of axial coding, CD and LW collaboratively merged the lists of both focus groups into one list, combining the decision variables which were similar in their meaning [32]. Subsequently, CD and LW translated the data into comprehensible decision variables in the English language. This translation was also an important interpretative step, as it required clinical education knowledge in addition to suggestions from the clinical teachers. There were only minor differences in interpretation and translation, which were resolved by discussion among the authors.

Next, to categorize the decision variables, we used the recently proposed [27] categories of *ability, integrity, reliability*, and *humility.*

## Results

The medical teacher focus group included eight clinical teachers (six males, two females, mean age 45.0 years, mean experience as clinical teacher 13.6 years); the veterinary teacher focus group comprised nine (3 males, 6 females, mean age 44.6 years, mean experience as clinical teacher 14.7 years). Both focus groups consisted of clinical teachers from various disciplines: within the medical focus group representatives from dermatology *(n* = 2), internal medicine (*n* = 2), ophthalmology (*n* = 1), otorhinolaryngology (*n* = 1), pathology (*n* = 1) and paediatrics (*n* = 1) were present. Within the veterinary focus group there were specialists in companion animal health (*n* = 3), equine health (*n* = 3) and large animal health (*n* = 3).

Data collection proceeded in both groups until theoretical sufficiency was reached concerning the current research question. The data were then critically reviewed by the authors for their unique nature. Whenever two decision variables were basically similar, they were combined into one item. This process resulted in a list of 11 and 17 decision variables from the medical and veterinary group, respectively, certified as relevant. The subsequent process of axial coding yielded a total of 21 unique decision variables mentioned as being relevant in one or both groups, as listed in Table 1.

**Table 1:** Decision variables needed to make valid entrustment decisions, ranked by the clinical teachers and divided into five categories

To make an entrustment decision about a learner performing a certain task, I need to …	Quotes	Ranking and points UMCU	Ranking and points FVMU
*Ability*			
Know the learner’s ability to use the right indication to execute a task	* ‘Someone should not only know how to execute a task, but also when to execute it and when not. (…) To tell the truth: the boundaries of the EPA. Is it still valid to execute this EPA.’ (FVMU)*	*(1) 31*	*(6) 5*
Know the learner’s theoretical knowledge level for the task	* ‘There has to be a certain level of knowledge about the preparation of a practical skill, and about the execution, aftercare and complications.’ (FVMU)*	*	*(1) 22*
Know the learner’s ability to explain the task to patients and other laymen	* ‘Being able to explain the task to a patient is really the litmus test to see if a learner gets the procedure.’ (UMCU)*	*(4) 11*	*(5) 7*
Know the learner’s experience with comparable, relevant tasks	* ‘I am thinking about a laparotomy or a caesarean section. If you can do a section perfectly, the laparotomy will also be executed fine, at least to some extent. (…) Talking about opening the abdomen would work.’ (FVMU)*	*	*(6) 5*
Know a learner’s ability to put a patient at ease during the task	* ‘They should have knowledge about the specific animal species they are handling. (…) Separately from the task itself. For example, doing a vena puncture is always with a vein and a needle, and the result is all the same, hopefully. But it differs how someone approaches the vena puncture with different species.’ (FVMU)*		*(6) 5*
Know the learner’s ability to handle risks and complications	* ‘I want to know whether a learner knows which risks there are and how to deal with these risks. What do you do when you take a biopsy and the wound starts to bleed or the patient faints?’ (UMCU)*	*(6) 4*	*
Know the learner’s organizational skills	* ‘To give an example: we have learners who can execute a task very well if you give them all the stuff in their hands and tell them what to do. But when you ask them: ‘please give this horse an injection’, it will cost them half an hour to organize everything. Organizational skills.’ (*FVMU*)*		*(8) 3*
Know the quality and accurateness of a learner’s written report about a task	* ‘An important thing to me, is a learner who can execute a task in such a way someone else can just take over. (…) It has to do with patient handover, about registration, working accurately, that kind of thing.’ (UMCU)*	*(8) 2*	*
Have information about the time span a learner needs to perform a task	*‘I was thinking … When someone takes a day for a task which can be performed in an hour …’ (FVMU)*		(9) 2
*Humility*			
Know the learner’s own judgement about his/her competence to execute the task	* ‘I would start by asking the learner: “Do you think * *you can do it on your own?”’ (UMCU)*	*(9) 1*	*(4) 9*
Know the learner will ask for supervision in time	* ‘I would really like to know the learner understands, even when he/she is allowed to execute a task independently, when he/she should call a supervisor. I like to call this: ‘call when panic’.’ (UMCU)*	*	*(7) 4*
*Integrity*			
Have a step-by-step dialogue with the learner about the execution of a task	* ‘In a situation when the learner does his thing, but with every step he deliberates with you, you can determine very well if the learner knows what to do and what the treatment plan would be and so on. (…) If he does a step, he discusses the step with you and he reflects if the step is sufficient or recognizes things are missing. (…)’ (FVMU)*		*(8) 3*
Know the learner is taking the patient’s welfare into account	* ‘Maybe to take animal welfare into account, and to base decisions on this.’ (FVMU)*		*(10) 1*
*Reliability*			
Know the learner is consistent and predictable in his or her behaviour	* ‘I want to have the trust a learner will execute this task the same way in the future.’ (UMCU)*	*(2) 22*	*(4) 9*
Know the learner’s ability to recognize and execute the task in deviating situations	* ‘Someone is good when he is able to get to the diagnosis in one straight line. If someone moves around this red line, then he is less entrustable, to say it like this. This has to do with the time (…), but also: which turnings does he take?’ (FVMU)*	*(7) 3*	*(4) 9*
Know the learner has situational awareness regarding a task	* ‘If people have to learn a task, they completely focus on the task (…). But there is a lot more around it: a time out procedure, the correct positioning of a patient, comforting the patient, (…), etcetera. Those things are part of it when you want to execute a task independently.’ (UMCU)*	*(6) 4*	
Know the learner’s professional behaviour	* ‘I would like to have information about the learner’s professional behaviour, when he executed a task (…) under supervision in the past.’ (FVMU)*		*(10) 1*
*Adequate exposure*			
Personally observe the task execution as often as needed	* ‘The proof of the pudding is in the eating: just show your skills to me.’ (UMCU)*	*(3) 19*	*(4) 9*
Know how often a task has been performed under which level of supervision and its results	* ‘I would like to know the amount of supervision the learner already has experienced on this task before I saw him for the first time.’ (FVMU)*	*(5) 9*	*(3) 15*
Know the judgement of other supervisors’	*‘What do other supervisors think of this learner? (…) Maybe a learner doesn’t totally execute the task the way I would like to see it done, but ten of my colleagues may think it is fine and give high marks.’ (UMCU)*	*	*
Have access to feedback or judgements of others (patients, patient-owners, family, nurses, paramedics, peers, other caretakers)	* ‘The most important thing is to know how a learner has been assessed. Not by one teacher, but by multiple teachers. Not by one peer, but by multiple peers. So, I would like to have information from multiple sources.’ (FVMU)*	*	*(2) 19*
Know a learner practised in a simulation situation	* ‘I would like to know if the skills needed for this task have already been practised in a dummy situation or other simulation situation and what the result of this practice was.’ (UMCU)*	*(9)1*	

*UMCU* University Medical Center Utrecht, *FVMU* Faculty of Veterinary Medicine, Utrecht

* also mentioned by the clinical teachers, but was not ranked by them in the focus group session

The decision variables were organized around five categories, four of which were recently proposed as a framework [27]: *ability, humility, integrity, reliability*, supplemented with a new category ‘*adequate exposure’*. In both groups 14 similar decision variables were mentioned, 2 decision variables were only mentioned by the medical group and 6 only in the veterinary group.

Most of decision variables were categorized as ‘ability’. The two highest ranked decision variables belonged to this category. Clinical teachers in the FVMU focus group mentioned ‘insight in the learner’s level of task-related knowledge’ as the most relevant variable. The most relevant decision variable for the medical group (UMCU) was ‘the learner’s ability to use the right indication to execute [a task]’. Furthermore, decision variables belonging to reliability and improvability were highly ranked by the participants. The decision variable ‘have knowledge about the judgement of other supervisors’ was the only variable mentioned but not ranked.

The categories *ability, integrity, reliability, *and* humility *are the conditions that must be met before someone trusts another person [7, 16, 19, 27]. Despite of this knowledge, our participants mentioned five variables that do not fit within these categories e. g.: *personally observing the task execution as often as needed* and *knowing how often a task has been performed under which level of supervision and its results* (see Table 1). These decision variables contain information about the learner based on similar or prior encounters. To categorize these variables we added a new category named ‘*adequate exposure’*.

## Discussion

This study aimed to increase insight into decision variables that clinical teachers apply when making entrustment decisions in the clinical workplace. In total 21 decision variables—divided over the categories *ability, humility, integrity, reliability* [27] and *adequate exposure*—were perceived as relevant for entrustment decision-making. These decision variables could help to build arguments for the multifactorial process of making an entrustment decision and deciding whether a learner is able to execute an EPA at a certain entrustment level.

Results from our study elaborate and expand on findings about entrustment decision-making in the literature [11, 16, 27], and add new insights and not yet listed decision variables. From previous research we know that decisions about supervision level are usually based on more than assessments of clinical skills [11, 16, 23, 27]. Clinical teachers assess the ‘trustworthiness’ of learners to act independently based on four categories: knowledge and skills, discernment, conscientiousness, and truthfulness [16]. We had to add a new category *adequate exposure; *these decision variables contain information about the learner based on similar or prior encounters, not specific about the learner or characteristics of the learner.

Previous studies have investigated variables needed to trust a learner to perform a critical task at a specific level of supervision [4, 16–19, 33]. To gain sufficient trust to perform a task at a specific level of supervision (direct supervision, indirect supervision, distant supervision or no supervision), learners need to demonstrate these qualities and skills. This trust cannot be credited to a single quality or skill, but requires a multitude of variables [4, 8]. Extensive assessment and feedback by different observers on performed tasks is highly relevant: trust is multifactorial and highly contextual, it occurs under the broad constructs of task, supervisor, learner, and environmental factors, and is well described in prior work [4]. In line with previously published evidence on workplace-based assessment, direct observation of a task by a teacher was perceived as relevant [23, 30]. On the other hand, this study indicates that extensive reflection by the learner and feedback from different observers on the performed tasks are highly relevant, also with respect to the formative function of EPAs.

The need for robust information for entrustment decision-making aligns well with current thinking about programmatic assessment [34–36]. In line with previously published evidence on workplace-based assessment, direct observation of a task by a teacher was perceived as relevant [30, 37]. On the other hand, this study indicates that extensive reflective behaviour by the learner and feedback from different observers on the performed tasks are highly relevant with respect to the formative function of EPAs.

A possible limitation of our study is that the focus groups were held in two schools of one university. On the other hand, the fact that it was held at both the veterinary and medical school broadens the generalizability of the results. Both groups of clinical teachers are from a similar (Dutch) educational culture, which is not necessarily transferable to other countries. The number of participants in each focus group was appropriate to allow for sufficient and balanced expression of views [28, 29]. Another limitation of this study could have been the trigger cases presented in the focus groups as they could have influenced the clinical teachers during the sessions. However, during the introduction of the trigger case, the moderator emphasized that this was just one example of an authentic clinical task and participants were explicitly encouraged to think beyond the trigger case.

The feasibility of the listed decision variables needs to be tested by their actual use in educational practice, i. e. whether teachers take these variables into account in entrustment decision-making. The variables could be used in ad-hoc entrustment decisions and as part of summative entrustment decisions. While we did not explicitly present this distinction during the focus groups, we found hesitation among participants to make critical decisions to grant responsibility when insufficient information was available [13]. Faculty development will be necessary to explain these types of entrustment decisions as being situational versus focused on summative assessment, when introducing workplace curricula based on EPAs [12, 14]; clearly in the latter case, our participants felt a need to have sufficient information to ground entrustment decisions. Further research could optimize the decision variables in the clinical workplace and could outline decision variables specific in ad hoc situations and variables that ground summative entrustment.

## Conclusion

This study aimed to increase insights about relevant decision variables which are or can be used by clinical teachers to make an entrustment decision about learners in the workplace. These decision variables can assist clinical teachers in the entrustment process by supporting the multifactorial process of making an entrustment decision. Our participants felt a need to have sufficient information to ground entrustment decision, thus faculty development will be necessary to explain entrustment decisions as being situational versus focused on summative assessment, when introducing workplace curricula based on EPAs. Faculty development will be necessary to explain these types of entrustment decisions as being situational versus focused on summative assessment when introducing workplace curricula based on EPAs.
